# Frailty Trajectories and Influencing Factors in Patients With Non-Hodgkin Lymphoma During Chemotherapy: Protocol for a Longitudinal Mixed Methods Study

**DOI:** 10.2196/76628

**Published:** 2025-10-03

**Authors:** Ruofei Du, Ying Zhang, Huimin Yang, Yating Du, Jin Li, Bingyan Zhang

**Affiliations:** 1 Lymphoma Ward, The First Affiliated Hospital of Henan University of Science and Technology, No. 24 Jinghua Road, Jianxi District, Luoyang, 471000, China, 86 18837950376; 2 Department of Nursing, Henan University of Science and Technology, Luoyang, China; 3 Department of Nursing, Luoyang Maternal and Child Health Hospital, Luoyang, China

**Keywords:** frailty, non-Hodgkin lymphoma, chemotherapy, longitudinal mixed methods study, protocol

## Abstract

**Background:**

High-intensity chemotherapy for non-Hodgkin lymphoma (NHL) has been shown to improve survival outcomes. However, its substantial toxicity may induce or exacerbate patient frailty, which is strongly associated with treatment interruptions, increased adverse effects, and reduced survival rates. Despite this, the dynamic progression of frailty and its specific contributing factors among patients with NHL undergoing chemotherapy remain poorly understood.

**Objective:**

This study aims to investigate the heterogeneity of frailty trajectories and explore their risk factors in patients with NHL. We seek to generate insights into the dynamic relationship between frailty and disease course. The findings may offer health care professionals dynamic insights into frailty progression and could help inform strategies for the early identification of high-risk populations through the systematic assessment of key factors. Ultimately, this work seeks to contribute to the evidence base for developing interventions that could mitigate or prevent frailty.

**Methods:**

This longitudinal mixed methods study will recruit 250 patients newly diagnosed with NHL from multicenter hospitals in China. Quantitative data will be collected at 3 time points: before chemotherapy, during the third cycle of chemotherapy, and at the end of chemotherapy. We will use validated questionnaires (ie, the Tilburg Frailty Indicator) to gather information on sociodemographics, frailty, cognition, physical condition, health literacy, anxiety, and nutrition. Qualitative data will be collected via semistructured interviews and observations at the end of chemotherapy. The growth mixture model and logistic regression analysis will be used to analyze quantitative data, and the diachronic analysis method and the directed content analysis method will be used for the qualitative data. Both types of data will be analyzed in parallel and separately. Finally, we will integrate the datasets to identify areas of confirmation, complementation, or discordance.

**Results:**

The research protocol and informed consent form were approved by the Medical Ethics Committee of the First Affiliated Hospital of Henan University of Science and Technology (2024-03-K171). Recruitment began in January 2025, with 168 participants enrolled as of September 2025. The data collection and analysis processes are expected to be finalized by March 2026. Data management is still ongoing; therefore, data analysis has not yet been conducted.

**Conclusions:**

As a pilot trial, this research is primarily designed to assess the feasibility of our methods and generate preliminary data on frailty progression. The findings could offer initial insights and help inform the development of future strategies for the early identification of high-risk populations through systematic screening, with the ultimate goal of informing interventions that may prevent frailty.

## Introduction

### The Frailty of Patients With Non-Hodgkin Lymphoma

According to global health statistics, approximately 544,352 new cases of non-Hodgkin lymphoma (NHL) were reported worldwide in 2020 [1]. Chemotherapy remains the frontline treatment for NHL, significantly improving cure and survival rates. However, factors including systemic chronic inflammation, recurrent fever, chemotherapy-induced adverse effects, and high disease relapse rates contribute substantially to the deterioration of physical and mental function in patients with NHL, thereby predisposing them to frailty [2]. Frailty is a clinical syndrome in which an individual’s physiologic reserve capacity and resistance are reduced, resulting in increased vulnerability to stressors and, consequently, a high risk of adverse health outcomes [3]. Frailty can lead to an increased risk of falls, infections, myelosuppression, prolonged hospitalization, and mortality [4]. A previous study reported that, among patients with NHL aged over 65 years, the prevalence of prefrailty was 62.1%, whereas the prevalence of frailty was 39.9% [5]. It is important to research how to alleviate frailty in patients with NHL.

However, most frailty studies are cross-sectional in nature. Previous studies have revealed a high incidence rate and several influencing factors, such as the Charlson Comorbidity Index, malnutrition, and impaired mobility [5]. However, cross-sectional studies evaluate data at a certain time point and cannot observe dynamic changes in frailty. Previous longitudinal studies [6] have only assessed frailty status before and after chemotherapy and reported the proportion of people with changes in frailty status but could not carefully observe the change trajectory of frailty over the course of chemotherapy and the influencing factors of different trajectories. It is essential to understand the longitudinal changes in patients with NHL. First, Gobbens et al [7] defined frailty as a dynamic process characterized by the interplay and mutual influence of multiple factors. Second, the National Comprehensive Cancer Network recommends that health care providers promptly identify, assess, monitor, and document the frailty of patients with cancer across all age groups and disease stages during their initial visit, routine cancer treatments, and posttreatment follow-up [8]. To the best of our knowledge, there is a notable lack of comprehensive longitudinal studies examining the frailty of patients with lymphoma throughout their chemotherapy treatment. A longitudinal study can systematically observe the progression of frailty in patients across various disease stages and types of lymphoma. Early identification of frailty can facilitate more effective prevention and management strategies, thereby providing health care professionals with valuable insights for delivering precise and targeted care.

### The Necessity of Longitudinal Mixed Research

Longitudinal mixed studies can help understand the experience and influencing factors of complex phenomena from multiple perspectives and reduce biases in research, and the results of dynamic trajectories are also generalizable. This is because frailty is a multidimensional concept involving physical, psychological, and social aspects [3]. Longitudinal quantitative studies tend to adopt predefined universal scales for objective measurement of various factors, failing to reflect the specific experiences and perceptions of patients. The incorporation of qualitative research can provide an in-depth understanding of the changing process of subjective experiences, such as personal histories, feelings, and needs of respondents, enhancing the depth and breadth of the research [9].

In summary, while the significance of the frailty trajectory and its influencing factors in patients with NHL has been highlighted, it remains unverified. Consequently, there is a pressing need for additional high-quality studies to address this gap. Such research may provide health care professionals with dynamic insights into frailty progression. Identifying key contributing factors could help inform strategies for the early identification of high-risk populations through systematic screening. Ultimately, this work could contribute to the evidence base for developing interventions aimed at frailty prevention. Furthermore, the findings might inform the development of relevant health policies.

### Aims

The study’s aims are as follows:

1. To explore and describe patterns of change in frailty scores over time in patients with NHL
2. To investigate potential predictors of frailty trajectories by integrating quantitative longitudinal data with qualitative insights

## Methods

### Theoretical Framework

The integral conceptual model of frailty (ICMF) and the health ecology model (HEM) will be used to guide this study. The ICMF was developed by Gobbens et al [7]. This model posits that frailty is influenced by a multitude of factors, including social demographics, chronic conditions, and lifestyle choices. This progression culminates in various forms of frailty—physical, psychological, and social—and results in adverse health outcomes such as illness, disability, and mortality. The previous research is reliable and reproducible [10]. Therefore, we use the ICMF to guide the identification and selection of factors influencing frailty in longitudinal quantitative research. Specifically, physical (ie, gender and age), psychological (anxiety), and social (marital status) factors contribute to the frailty of patients with NHL, and both these factors and patients’ frailty status evolve dynamically throughout the chemotherapy cycles.

The HEM underscores the intricate interplay between environmental factors and human health. The HEM has been extensively applied in guiding mixed research on influencing factors [11], and the results of these studies are good. On the basis of each of the 5 dimensions of the HEM (personal traits, living and working conditions, psychological and behavioral characteristics, interpersonal network, and macro factors), we developed an interview framework to explore the risk factors of frailty and integrate the findings of qualitative and quantitative data.

**Figure 1.**
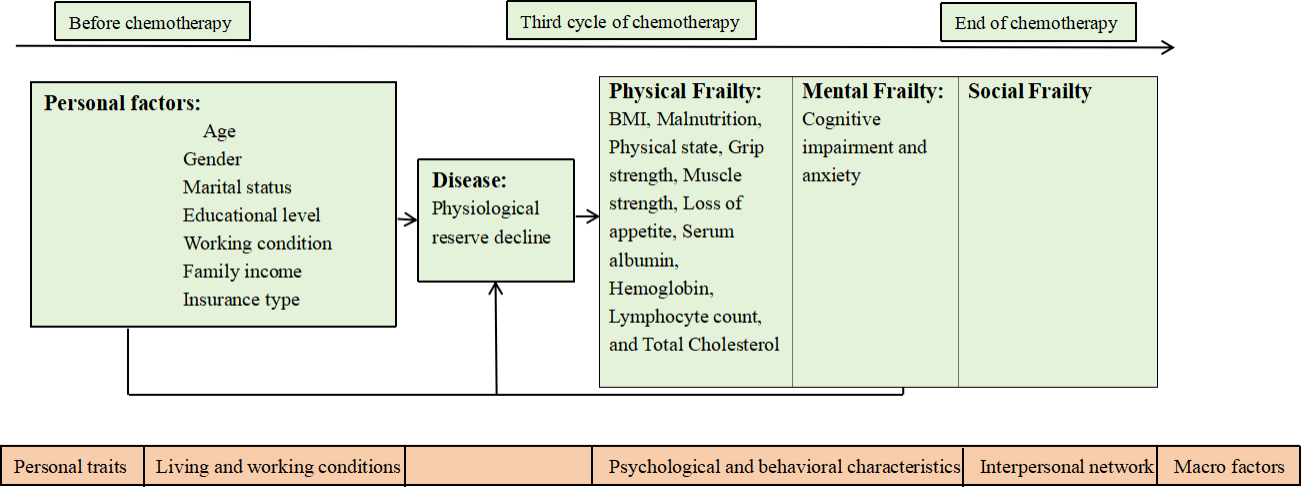
Theoretical framework of the study.

The interaction between the ICMF and HEM will be implemented during the mixed methods integration phase using iterative joint displays. These tables will serve as an analytical tool to directly facilitate dialogue between the 2 models. The process consists of 2 steps. In step 1 (quantitative data driven), a joint display will be developed to examine a key quantitative finding (ie, “unmarried patients [ICMF social domain] exhibit significantly worse frailty trajectories”). The left column of this display will present this quantitative result, whereas the right column will provide qualitative excerpts from participant interviews illustrating lived experiences of social support (HEM interpersonal network dimension) and its interaction with other ecological levels (ie, HEM organizational level: access to support groups; HEM community level: transportation barriers affecting socialization). Conversely, in step 2 (qualitative data driven), a prominent theme from qualitative analysis (ie, “the pervasive experience of financial toxicity influencing treatment decisions,” an HEM community and policy construct) will be placed in the left column of the joint display. The right column will then display quantitative data (ie, income levels, comorbidity scores, or medication adherence rates from the ICMF biological domain) to demonstrate the prevalence of this experience and correlate it with measurable outcomes. The theoretical framework for this study is built by combining these 2 theories (Figure 1).

### Study Design

**Figure 2.**
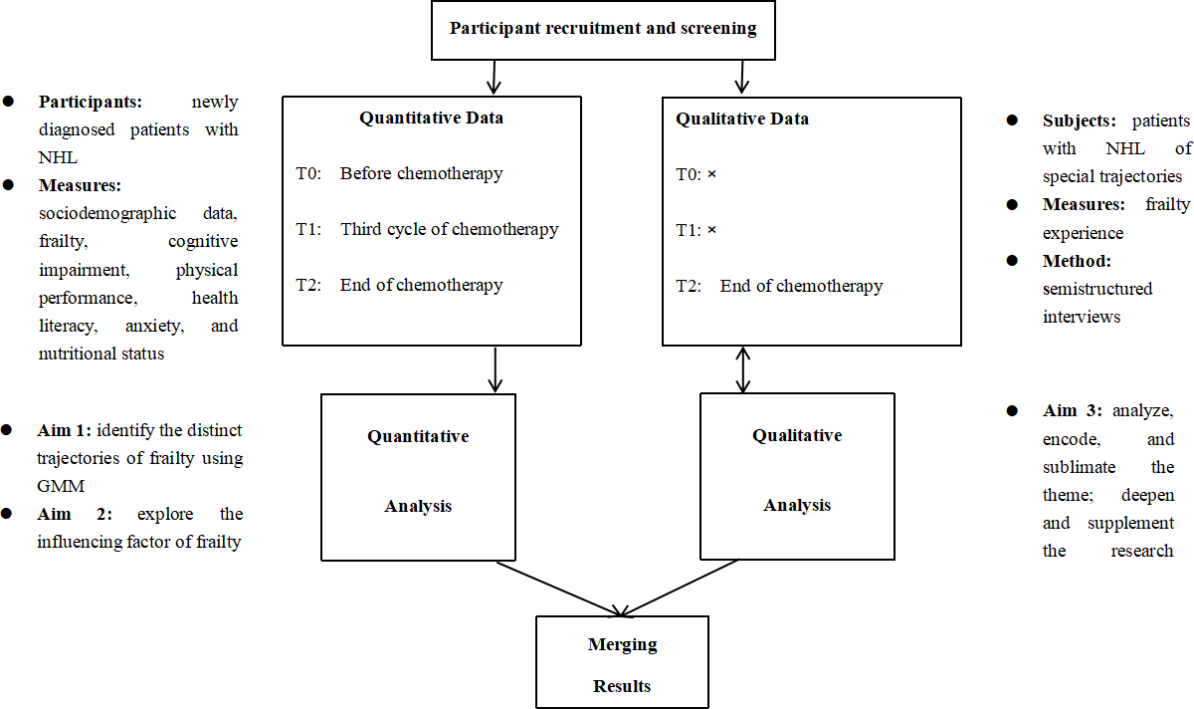
Flowchart of the longitudinal mixed methods study design. GMM: growth mixture model; NHL: non-Hodgkin lymphoma.

**Figure 3.**
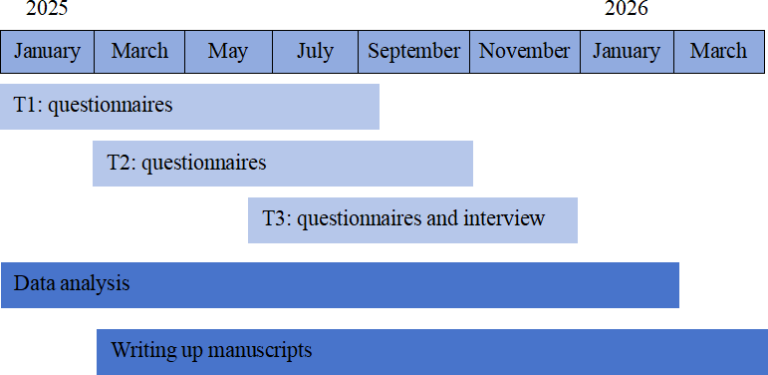
Project timeline.

A longitudinal mixed methods approach will be used in this study. This study intends to conduct separate quantitative and qualitative analyses. First, case information, somatic measurement data, and questionnaire responses will be collected from patients who meet the specified inclusion criteria at multicenter hospitals in China. The heterogeneity of trajectories and influencing factors will be identified through rigorous quantitative analysis. Second, in-depth qualitative interviews will be conducted with patients whose frailty trajectories exhibit significant changes and a tendency toward deterioration, yielding qualitative insights into factors contributing to frailty. Finally, the quantitative and qualitative data will be integrated in this study to conduct a more targeted mixed analysis. Figure 2 shows a diagram of the longitudinal mixed methods design. The survey began in January 2025 and is scheduled to end in March 2026 (Figure 3).

### Quantitative Component

#### Data Collection

Patients who have been newly diagnosed with NHL will be recruited from the department of hematology of 5 large public hospitals in China via a multicenter, consecutive convenience sampling strategy. This method was selected for its feasibility, cost-effectiveness, and practicality in clinical settings. To reduce selection bias and enhance rigor, the following measures will be taken. All eligible patients at participating sites will be invited sequentially during the enrollment period to avoid preferentially recruiting more motivated or healthier individuals. Strict predefined inclusion and exclusion criteria will be applied to maintain a homogeneous study sample. Each site will keep a detailed log tracking the number of patients screened, those who decline, and their reasons. This will enable transparent assessment of recruitment and potential nonparticipation bias. Comprehensive baseline demographic and clinical data will be collected for all participants, allowing for comparison with the broader target population and establishing the generalizability of the findings.

The National Comprehensive Cancer Network Clinical Practice Guidelines in Oncology: Lymphoma (2024 edition) [12] indicate that, if a patient responds effectively to treatment, the efficacy evaluation may demonstrate either a complete or partial response within 2 to 4 cycles of chemotherapy. Following the completion of chemotherapy, the disease can remain stable for an extended period. Therefore, the time points of this longitudinal study are before chemotherapy (T1), the third cycle of chemotherapy (T2), and the end of chemotherapy (T3). Prechemotherapy measurement (baseline) captures frailty status before treatment, helping distinguish the disease’s effect from treatment-related toxicity. Measuring after the third cycle of chemotherapy (T2) captures acute toxicity—such as fatigue or neuropathy—when side effects are most severe and frailty often increases. The T3 measurement (after treatment completion) helps identify recovery in responders or persistent frailty in nonresponders, revealing medium-term outcomes. All eligible patients will be identified via the hospital’s case management system and invited to participate within 24 hours of admission. Researchers will administer standardized questionnaires in accordance with the same guidelines either in the ward meeting room or at the participants’ preferred location. Data collection will be conducted by 2 researchers, who will subsequently collate and archive the data. To ensure quality control, the following methods will be used in this study. First, unified training will be conducted for the implementation members of the research group. The principal researcher will elaborate on the content and significance of each item of the questionnaire and inform them of the precautions in the process of questionnaire administration. Second, theoretically, the questionnaire should be filled out independently by the research participants. If the research participants fail to understand or are unable to fill the questionnaire out by themselves, the researchers should communicate fully with them and fill it out objectively and truthfully on their behalf. Third, after the completion of the questionnaire, the investigators should collect it immediately and check and verify it promptly to ensure completeness and quality. We will strictly follow the requirements of the CONSORT (Consolidated Standards of Reporting Trials) statement to record the flow of participants throughout the research process, and provide a complete CONSORT flowchart in the final research paper.

#### Population and Sampling

The interaction effect repeated-measure analysis in the G*Power software will be used to calculate the required sample size, with each study participant measured 3 times. Under the conditions of an average correlation coefficient ρ of 0.5, power (1–β) of 0.8, significance level α of .05, and effect size *f* of 0.14 (to align with reported effect sizes [*f*=0.10-0.25] from related cancer studies [13], the chosen value [*f*=0.14] is a conservative, small effect size to ensure that we can detect even modest clinical changes), the initial calculated sample size was 105 participants. In addition, this study aims to adopt the Bayesian information criterion (BIC) of the growth mixture model as the primary model evaluation index [14]. Considering a follow-up loss rate of 20%, and to meet the requirement of N≥200 for BIC applicability, the final sample size should be at least 250 participants.

The inclusion criteria are as follows: (1) age of ≥18 years; (2) confirmed histopathological diagnosis of NHL; (3) treatment with cyclophosphamide, hydroxydaunorubicin, oncobin, and prednisone or with rituximab, cyclophosphamide, hydroxydaunorubicin, oncobin, and prednisone chemotherapy regimens; and (4) absence of severe cognitive impairment or communication disorders.

The exclusion criteria are as follows: (1) previous history of chemotherapy in conjunction with comorbid conditions and (2) history of psychiatric illness or use of psychotropic medications.

The shedding criteria are as follows: (1) voluntary withdrawal from the study and (2) chemotherapy-related incidents resulting in termination of treatment or patient death.

#### Study Measures

##### Overview

The variables in this study were chosen based on the literature, theoretical research frameworks, and expert consultation. Through a systematic review and meta-analysis, the research team concluded that the incidence rates of lymphoma, leukemia, and hematologic malignancies were 31.8%, 18.45%, and 10.4%, respectively, and identified some influencing factors of frailty (ie, gender, age, physical capabilities, general health condition, severity of disease, neurological and mental status, and biomarkers). By integrating the 3 dimensions of physical, psychological, and social frailty, we established that (1) the study population would comprise individuals with NHL and (2) the predictors would include sociodemographic factors, disease characteristics, physical condition, hand grip strength, nutritional status, mild cognitive impairment (MCI), and anxiety. The instruments and measurement times used in this study are shown in Table 1. The specific scales used are outlined in the following sections.

**Table 1.** Instruments and measurement times.

Instrument	Measurement time		
	Before chemotherapy	Third cycle of chemotherapy	End of chemotherapy
Sociodemographic questionnaire	✓		
Tilburg Frailty Indicator	✓	✓	✓
Montreal Cognitive Assessment–Basic	✓	✓	✓
Short Physical Performance Battery	✓	✓	✓
Chinese Version of the All Aspects of Health Literacy Scale	✓	✓	✓
Generalized Anxiety Disorder Scale	✓	✓	✓
Controlling Nutritional Status Score	✓	✓	✓

##### Sociodemographic Questionnaire

The comprehensive information questionnaire encompasses the following components. Personal factors include gender, age, marital status, and educational attainment. Disease-related factors include disease stage, comorbidities, grip strength, muscle strength, serum albumin levels, hemoglobin concentration, white blood cell count, C-reactive protein levels, and lymphocyte count. Among them, muscle strength was assessed by measuring the isometric strength of flexion in the dominant elbow joint. Grip strength was evaluated using a CAMRY electronic hand dynamometer (model EH101). The maximum grip strength value obtained from either hand was recorded as the representative measure. Low grip strength was defined according to sex-specific cutoffs: <30.2 kg for men and <17.2 kg for women.

##### Frailty

The Tilburg Frailty Indicator (TFI) will be used to evaluate frailty status [15]. The scale is a self-report questionnaire designed for patients, encompassing physical, psychological, and social dimensions. It consists of a total of 15 items. Each item uses a binary scoring method, resulting in a total score ranging from 0 to 15 points. A score of 5 or higher indicates frailty, with higher scores reflecting more severe levels of frailty. This scale has good reliability (Cronbach α>0.75) and validity.

##### Cognition

The cognition of patients will be measured via the Montreal Cognitive Assessment–Basic [16]. The assessment comprises 10 categories: executive function (1 point), immediate recall (0 points), fluency (2 points), orientation (6 points), computing ability (3 points), abstract thinking (3 points), delayed recall (5 points), visual perception (3 points), naming (4 points), and attention (3 points), totaling 30 points. For individuals with less than 6 years of education, a score between 13 and 19 indicates MCI. Those with 7 to 12 years of education are diagnosed with MCI if they score between 15 and 22 points. Individuals with more than 12 years of education require a score between 16 and 24 points for an MCI diagnosis. Previous studies have shown that the Montreal Cognitive Assessment–Basic has a sensitivity of 81% to 97% and a specificity of 60% to 86% for screening for MCI [17].

##### Physical Condition

The Short Physical Performance Battery will be used to measure physical condition [18]. The assessment comprises 3 components: a balance function test, a walking speed test, and a sit-to-stand test on a chair. Each component is evaluated on a scale from 0 to 4, yielding a total score ranging from 0 to 12. A total score of less than 10 indicates motor function abnormalities, with good reliability (Cronbach α>.85) and validity [19].

##### Health Literacy

The Chinese version of the All Aspects of Health Literacy Scale will be used to measure the health literacy of patients [20]. The assessment encompasses 3 dimensions and 11 items, including the ability to use written health information, the capacity to communicate effectively with health care providers, and the ability to evaluate and apply health information. The overall health literacy is categorized as high (>24 points), moderate (21-24 points), or low (<21 points), with good reliability (Cronbach α>0.79) and validity.

##### Anxiety

The Generalized Anxiety Disorder scale assesses the patients’ anxiety levels over the previous 2 weeks [21]. It comprises 7 items, with scores categorized as follows: a score of 0 to 4 indicates no anxiety, a score of 5 to 9 indicates mild anxiety, a score of 10 to 14 indicates moderate anxiety, and a score of 15 to 21 indicates severe anxiety, with good reliability (Cronbach α>0.859) and validity. The sensitivity and specificity were 84.31% and 91.67%, respectively.

##### Nutrition

The Controlling Nutritional Status score will use 3 indicators to screen for early malnutrition: serum albumin, total cholesterol level, and total lymphocyte count [22]. The scoring system is as follows: normal (score 0-1), mild malnutrition (2-4), moderate malnutrition (5-8), and severe malnutrition (9-12). The score has been widely used in nutritional screening for common malignant tumors [23].

### Strategies to Minimize Participant Burden and Reduce Attrition

This study involves collecting data for 66 items. Of these, 12 will be retrieved from the hospital’s medical record system, 15 will be measured by the research team, and only 33 will require direct input from patients. To reduce participant burden, the following measures will be implemented. First, all questionnaires will be administered during patients’ regular follow-up visits to avoid extra hospital trips. Second, to ensure that the measurement time does not exceed 20 minutes, this study will adopt a modular and segmented design for the reorganized questionnaire order. For instance, physical measurements such as BMI, grip strength, balance, gait speed, and chair stands will be grouped together. Third, a low-intrusion communication plan (ie, short reminder calls between assessments) will be used to sustain engagement without causing inconvenience. Fourth, if a participant cannot finish all measures, priority will be given to key instruments (ie, the TFI) to preserve essential data. Finally, reasons for dropout will be carefully documented. Should dropout rates exceed expectations, the team will meet to develop further strategies.

### Quantitative Data Analysis

The data will be imported into SPSS Statistics (version 26.0; IBM Corp) and R software (R Foundation for Statistical Computing) for comprehensive statistical analysis, and the test level will be treated according to a bilateral α of .05.

Different descriptive statistical methods will be used based on the types of data collected. For continuous variables that follow a normal distribution, the mean and SD will be used. Categorical variables will be summarized using frequency distributions and percentages. The frailty scores of patients with NHL at various time points will be treated as continuous variable data.

The participants who complete follow-up will be compared with those who are lost to follow-up. Continuous variables will be analyzed via independent-sample *t* tests (2-tailed), whereas categorical variables will be assessed via chi-square tests and the Fisher exact probability method. Repeated-measure ANOVA will be initially conducted to examine the frailty scores of patients with NHL across different time points, determining whether there are significant differences during various periods. This analysis will also provide a preliminary evaluation of the overall trend in frailty scores.

The growth mixture model will be used to analyze the potential categories of the trajectory. Initially, a single-class model will be constructed, followed by incrementally increasing the number of classes. The fit indexes of successive models will be compared to determine the optimal model considering both practical significance and statistical criteria. The goodness-of-fit indicators will include, first, the information criteria: the Akaike information criterion, BIC, and sample size–adjusted BIC. Lower values indicate better model fit. Second, likelihood ratio tests and bootstrap-based likelihood ratio tests will compare the K–1 and K-class models based on differences in likelihood ratios. A *P* value of <.05 suggests that the K-class model is statistically superior to the K–1 class model. The third indicator is entropy. This metric evaluates classification accuracy on a scale from 0 to 1, with higher values indicating greater precision. An entropy value of 0.80 indicates a classification accuracy exceeding 90%.

Chi-square tests and the Fisher exact probability method will be used for univariate analysis of categorical variables, whereas one-way ANOVA will be used for continuous variables to identify statistically significant indicators and conduct a preliminary analysis of differences among various trajectory categories within the datasets. Multivariate logistic regression analysis will be subsequently conducted, with the final determined frailty trajectory types of patients with NHL serving as the dependent variable. Indicators that were statistically significant in the univariate analysis will be included in the regression model for further analysis. The significance level α will be set at .05 to ultimately determine the influencing factors of frailty trajectories in patients with different types of NHL.

### Qualitative Component

#### Data Collection

This study will use the descriptive phenomenological research method. A semistructured interview approach will be used. The interviews will be scheduled in advance with the participants and take place in a quiet conference room to ensure privacy and prevent interruptions. The interviewees will be encouraged to articulate their perspectives and experiences comprehensively. We will use appropriate probing questions, paraphrasing, and summarization. In addition, nonverbal behaviors such as facial expressions and the body language of the interviewees will be observed and documented. Each participant’s interview will last approximately 30 to 40 minutes. The interview questions will be developed initially based on the HEM, existing research results, and experts' assistance. The interview guides will be constantly updated to adapt to the situations encountered during the research process. Within 24 hours following the conclusion of each interview, the recorded content will be transcribed into textual data. The transcription will include annotations of the interviewees’ basic information, recording durations, and interview time stamps. To safeguard the privacy of the respondents, their names will be anonymized via numerical identifiers. In the process of conducting the interviews, the following points will be focused on: (1) the content of later repeated interviews will be largely driven by the results of the previous interviews, and (2) this study will focus on “change”; that is, participants will reflect on the evolution of their frailty experience throughout the course of chemotherapy, as well as the factors that influenced these changes. The interview outline is shown in Textbox 1.

Textbox 1. **Interview outline.**

1. During the therapeutic process, did you have any sense of frailty and what was the severity level?
2. Has the sense of frailty changed from the start of the treatment until now?
3. During the therapeutic process, which aspects of your individual health status do you think could have influenced the aforementioned state and has the degree of influence changed?
4. During the therapeutic process, which behavioral traits or habits do you think could have influenced the aforementioned state and has the degree of influence changed?
5. During the therapeutic process, which aspects of interpersonal relations or interactions do you think could have influenced the aforementioned state and has the degree of influence changed?
6. During the therapeutic process, which aspects of living or working conditions do you think could have influenced the aforementioned state and has the degree of influence changed?
7. During the therapeutic process, which aspects of macro factors (such as economy, policy, and culture, etc.) do you think could have influenced the aforementioned state?

#### Population and Sampling

To alleviate the burden on participants and enhance the efficiency of this study, we will use a targeted sampling strategy. Specifically, interviews will be conducted with patients who exhibit significant variability in their frailty status and frailty duration based on quantitative research findings. The sample size will be determined when data saturation is achieved, meaning that no new themes emerge from the interviews.

#### Qualitative Data Analysis

The text materials will be imported into the NVivo software (version 12.0; Lumivero), and 2 individuals will independently conduct repeated reading, sorting, and coding of the imported materials. This study will be guided by the HEM and use the diachronic analysis method and the directed content analysis method. The specific steps are as follows: (1) determine the analysis unit, taking the sentences related to the time-varying influencing factors of frailty expressed by the patients with NHL as the minimum analysis unit; (2) thoroughly read the original materials multiple times and carefully study the text content; (3) develop a classification outline based on the HEM, taking personal traits, psychological and behavioral characteristics, interpersonal networks, work and living conditions, and macro factors as names for theme coding; (4) conduct content coding and classification, mark the significant connotations and concepts in the text content, classify the relevant content into the corresponding theme categories, and further analyze and classify the content to form subthemes; and (5) interpret and explain the results, establish the connection between the text content and the extracted themes and subthemes, and find the corresponding text examples from the original materials. Any discrepancies concerning data coding and subject classification will be systematically addressed through regular discussions. All study data will be meticulously retained to ensure their validity and integrity.

### Integrating Quantitative and Qualitative Data

A convergent parallel mixed methods approach will be used, wherein quantitative and qualitative data will be collected and analyzed independently. The influencing factors will be synthesized during the result interpretation phase to comprehensively explore the determinants of frailty in patients with NHL. Specifically, the factors impacting the frailty trajectory of these patients will be examined separately. Multiclassification logistic regression will be used to understand the factors affecting the subgroup classification of frailty trajectories. In addition, an in-depth study will be conducted on the frailty experience of patients with NHL and the descriptive characteristics of the influencing factors. The results will be synthesized into a unified, side-by-side comparative display. The integration of quantitative and qualitative data will be achieved through the development of joint display tables. These tables will visually juxtapose the key quantitative findings and qualitative themes side by side for each research question. This will allow for a systematic comparison. First, we will analyze consistent or convergent findings. When quantitative and qualitative findings converge and are mutually corroborated, we will interpret this as strong evidence for a robust conclusion. The qualitative data will provide rich, contextualized examples to illustrate the statistical trends. Second, we will analyze complementary findings. When findings of one method elaborate, clarify, or provide new dimensions to the findings of the other method, we will use them to build a more nuanced and comprehensive understanding of the phenomenon. Third, we will analyze discordant or inconsistent findings. Crucially, any instances of discordance will be treated as valuable opportunities for deeper analysis. We will actively investigate potential explanations for these discrepancies by re-examining the data. Possible reasons may include, first, temporal differences. Quantitative measures capture a snapshot in time, whereas qualitative interviews may reflect experiences over the entire chemotherapy cycle. A second possible reason is the level of analysis. Quantitative data may reflect group-level trends, whereas qualitative data may reveal individual-level exceptions or unique experiences. A third possible reason is response bias (social desirability bias in surveys [quantitative] vs anonymity and depth in interviews [qualitative]). The fourth possible reason is measurement versus meaning. Numbers measure magnitude, whereas narratives capture meaning and context, which may not always align directly. We will report these discordances transparently and use them to generate new hypotheses or propose refined explanations that account for the complexity of the data.

### Feasibility Considerations

To ensure the feasibility of the mixed methods approach within the oncology setting, the following strategies will be implemented. Quantitative and qualitative data collection will be staggered to avoid burdening participants, with interviews conducted flexibly (in person or virtually). Our research team will receive specialized training for working with an oncology population. Finally, a pilot phase will test and refine the integrated protocol’s flow and acceptability.

### Patient and Public Involvement

Patients and the public were actively engaged in the design process for selecting the frailty scale, follow-up time points, result analysis, and dissemination. First, 5 patients with NHL of diverse ages, genders, and educational backgrounds were recruited to complete the TFI, the G8 questionnaire, and the Groningen Frailty Indicator. Among them, 4 patients found the TFI to be straightforward and comprehensive in capturing the multidimensional aspects of frailty, whereas 1 patient preferred the simplicity of the G8 questionnaire. On the basis of these patient insights, the TFI was ultimately selected as the primary tool for assessing frailty. Second, semistructured interviews were conducted with patients who had completed their chemotherapy regimens. These revealed that most patients experienced significant physical frailty and fatigue during the third cycle of chemotherapy and exhibited pronounced psychological frailty at the time of diagnosis. Consequently, we determined that follow-up assessments would take place before the initiation of chemotherapy, during the third cycle, and upon completion of the treatment. During the phase of result analysis, they will sense check our interpretations and help prioritize findings from a patient perspective. We will partner with patient and public involvement members in the dissemination phase through co-designing lay summaries, developing accessible materials, and offering opportunities for copresentation.

### Ethical Considerations

The study, research protocol, and informed consent form were approved by the Medical Ethics Committee of the First Affiliated Hospital of Henan University of Science and Technology in December 2024 (2024-03-K171), and the study will be conducted in accordance with the Declaration of Helsinki.

To rigorously ensure comprehension and voluntariness among patients who may be experiencing frailty, cognitive impairment, or treatment-related distress, we will implement an enhanced consent process. This involves explaining the study using a simplified consent form followed by a structured verification of understanding. Using the teach-back method, we will ask participants to explain key aspects in their own words (ie, “Please tell me what you believe the main purpose of this study is”). We will also directly assess their understanding of the core elements of consent: the study’s purpose, procedures, and risks and their right to withdraw without any impact on their clinical care. This dialogue will continue iteratively until clarity is achieved. Consent will be sought in a private setting to minimize pressure and will be reaffirmed as an ongoing process at subsequent visits. Participants will receive a $10 comfort package containing a soft blanket, a high-quality water bottle, and hypoallergenic lip balm as a token of appreciation for their participation.

During the study, the collection, use, and disclosure of research information will be conducted strictly in confidence and authorized in accordance with regulatory requirements. Patient information will not be disclosed outside the research center to ensure confidentiality. To safeguard the integrity of the study participants’ information, data will be stored in a segregated database accessible only by authorized researchers. Qualitative study records will be transcribed anonymously to protect participant identity. There are no anticipated ethical, legal, or security concerns associated with the collection, storage, or dissemination of any data related to this research. The findings of this study will be shared with academic, clinical, and public audiences to broaden their impact. Regarding the academic audience, results will be published in an international peer-reviewed journal following CONSORT guidelines and presented at nursing conferences. Regarding the clinical audience and participants, plain-language summaries will be provided to participating hospitals and staff, with results offered for presentation at clinical meetings. Participants will also receive a summary if they consent. Regarding public and patient engagement, key results will be shared via social media. In line with SPIRIT (Standard Protocol Items: Recommendations for Interventional Trials) guidelines, anonymized data and study documents will be made public on Figshare within 12 months of publication (ie, accessible to researchers with an approved proposal and data sharing agreement).

## Results

Participant recruitment began in January 2025. The number of recruited participants was 168 in September 2025. The data collection and analysis processes are expected to be finalized by March 2026. Data management is still ongoing; therefore, data analysis has not yet been conducted. The results are expected to be published in August 2026.

## Discussion

### Anticipated Findings

To the best of our knowledge, this is the first longitudinal study of frailty among patients with NHL. First, this study deepens the application of the frailty integration model to a longitudinal study. Previous research has established the efficacy of the ICMF in cross-sectional studies [24]. However, an outstanding feature of the ICMF is that the processes leading to frailty change dynamically over time [15]. Second, we investigate the application and validation of the longitudinal mixed methods approach in the context of frailty, thereby expanding the scope of research within this domain. Frailty is a multidimensional syndrome encompassing physical, psychological, and social dimensions and is influenced by a myriad of factors. The use of longitudinal mixed methods research can increase both the depth and breadth of our understanding of this complex condition. Finally, this study will explore and describe patterns of change in frailty among patients with NHL. This study will also identify the potential predictors of frailty in patients. This study will take a longitudinal mixed methods approach to collect and integrate quantitative and qualitative data. By combining the benefits of these 2 methods, this study uses statistical analysis and data mining techniques alongside in-depth interviews and observational methods to conduct a comprehensive and rigorous examination of frailty among patients undergoing chemotherapy. This approach seeks to thoroughly investigate influencing factors and explore the underlying mechanisms to facilitate the early identification of at-risk patients by health care professionals. The findings are expected to inform the development of targeted nursing interventions and provide a stronger evidence base for dynamic clinical decision-making.

However, we acknowledge several potential methodological threats and outline proactive strategies to mitigate them. Participant dropout may introduce bias. To minimize attrition, we will use flexible scheduling, maintain regular contact through check-in calls, and offer compensation. All dropout reasons will be meticulously recorded and analyzed. Inconsistency in data collection across sites is a risk. To ensure uniformity, all research staff will undergo centralized, standardized training using a detailed manual of procedures. Ongoing quality assurance checks will be conducted. Participants’ recollections in qualitative interviews may be imperfect. To enhance accuracy, interviewers will use timeline anchoring techniques with visual aids and ask probing questions focused on specific events and experiences.

### Strengths and Limitations of This Study

This research has the following strengths. The first strength is that a longitudinal mixed methods approach will be used to explore frailty in patients with NHL from both quantitative and qualitative perspectives. The second strength is that this is the first multicenter, mixed methods study conducted in China to investigate the heterogeneity and correlations of frailty in patients with NHL. The third strength is that our longitudinal study design will enable us to observe the trajectory and risk factors associated with changes in frailty among patients with NHL over time. This study will provide dynamic information for future frailty interventions and policy formulation. The limitations of this study are the potential loss of follow-up, inconsistency in data collection across sites, and recalling bias.

## Abbreviations

BIC: Bayesian information criterion
CONSORT: Consolidated Standards of Reporting Trials
HEM: health ecology model
ICMF: integral conceptual model of frailty
MCI: mild cognitive impairment
NHL: non-Hodgkin lymphoma
SPIRIT: Standard Protocol Items: Recommendations for Interventional Trials
TFI: Tilburg Frailty Indicator
